# *Salmonella* pSLT-encoded effector SpvB promotes RIPK3-dependent necroptosis in intestinal epithelial cells

**DOI:** 10.1038/s41420-022-00841-9

**Published:** 2022-02-02

**Authors:** Kedi Dong, Yuan Zhu, Qifeng Deng, Lanqing Sun, Sidi Yang, Kai Huang, Yu Cao, Yuanyuan Li, Shuyan Wu, Rui Huang

**Affiliations:** 1 Department of Medical Microbiology, School of Biology & Basic Medical Sciences, Medical College of Soochow University, No. 199, Ren Ai Road, Suzhou, Jiangsu 215123 P. R. China; 2 Centre for Infection and Immunity Studies (CIIS), School of Medicine, Shenzhen Campus of Sun Yat-sen University, Shenzhen, Guangdong 518107 P. R. China; 3 Cambridge-Suda Genomic Resource Center, Jiangsu Key Laboratory of Neuropsychiatric Diseases, Medical College of Soochow University, Suzhou, Jiangsu 215123 P. R. China

**Keywords:** Infection, Necroptosis, Antimicrobial resistance

## Abstract

*Salmonella* is one of the most important worldwide zoonotic pathogens. After invading a host orally, the bacteria break through the intestinal epithelial barrier for further invasion. Intestinal epithelial cells (IECs) play a crucial role in maintaining the integrity of the intestinal epithelial barrier. Necroptosis is considered one of the virulence strategies utilized by invasive *Salmonella*. Our previous work has shown that SpvB, an effector encoded by *S*. Typhimurium virulence plasmid (pSLT), promotes bacterial translocation *via* the paracellular route. However, it is still unknown whether SpvB could promote bacterial invasion through disrupting the integrity of IECs. Here, we demonstrated that SpvB promoted necroptosis of IECs and contributed to the destruction of the intestinal barrier during *Salmonella* infection. We found that SpvB enhanced the protein level of receptor-interacting protein kinase 3 (RIPK3) through inhibiting K48-linked poly-ubiquitylation of RIPK3 and the degradation of the protein in an autophagy-dependent manner. The abundant accumulation of RIPK3 upregulated the phosphorylation of MLKL, which contributed to necroptosis. The damage to IECs ultimately led to the disruption of the intestinal barrier and aggravated infection. In vivo, SpvB promoted the pathogenesis of *Salmonella*, favoring intestinal injury and colonic necroptosis. Our findings reveal a novel function of *Salmonella* effector SpvB, which could facilitate salmonellosis by promoting necroptosis, and broaden our understanding of the molecular mechanisms of bacterial invasion.

## Introduction

*Salmonella* is a common foodborne pathogen that poses an urgent health-safety problem. The species of *Salmonella* are highly diverse, and various serovars have different host specificity and clinical symptoms [1]. While most serovars cause mild gastroenteritis, some serovars in individuals with weakened immune systems can lead to severe and invasive infections, such as enteric fever and invasive nontyphoidal *Salmonella* disease, resulting in long-term health consequences and even death [2, 3]. *Salmonella enterica* serovar typhimurium (*S*. Typhimurium) is one of the most common isolates that can infect both humans and animals. A comprehensive understanding of the molecular mechanisms of *S*. Typhimurium is crucial for expanding therapeutic strategies against infectious diseases.

After breaking through the mucosal epithelial barrier, *S*. Typhimurium is mainly engulfed by immune cells such as macrophages. *S*. Typhimurium can replicate within phagocytic cells, facilitate further bacterial colonization, and cause systemic infection. A wide range of virulence determinants and effectors encoded by these genes are crucial for *S*. Typhimurium to invade the host and cause pathological changes [4]. Among them, *Salmonella* plasmid virulence (*spv*) is a highly conserved 8-kb-long region located on pSLT, which is crucial for intracellular survival and growth [5]. The *spv* gene consists of the positive regulatory *spvR* gene and the four structural *spvABCD* genes. Genetic analysis has demonstrated that the *spvB* gene contributes to the pathogenesis of *Salmonella* infection [6]. As shown in our previous work, *spvB-*encoded effector SpvB disrupts epithelial intercellular junctions, which is conducive to the paracellular translocation of bacteria across the intestinal epithelial barrier [7]. The intestinal epithelial barrier consists of IECs and junctional complexes [8]. In addition to the involvement of paracellular translocation, it remains to be clarified whether SpvB could promote *S*. Typhimurium invasion by disturbing the integrity of IECs.

*S*. Typhimurium infection generally causes obvious intestinal injury and cell death. Even though necrotic cell death was long defined as a form of unregulated and uncontrollable accidental cell death, recent studies have reported that a type of regulated cell death (RCD) shows morphological features similar to necrosis, termed necroptosis. Necroptosis has been implicated in many pathologies, such as inflammatory bowel disease. It is triggered by the dysregulation of either extracellular or intracellular homeostasis and requires the activity of mixed-lineage kinase domain-like protein (MLKL) and receptor-interacting protein kinase 3 (RIPK3). Phospho-MLKL (p-MLKL) mediates pore formation, which is a molecular basis for this lytic form of programmed necrosis [9]. In this study, we reported a novel function of the effector SpvB to disrupt the integrity of the intestinal epithelial barrier by inducing the necroptosis of IECs, thus promoting the invasion of *Salmonella*.

### Result 1 *Salmonella* pSLT-encoded effector SpvB induces caspase‐independent cell death

**Fig. 1:**
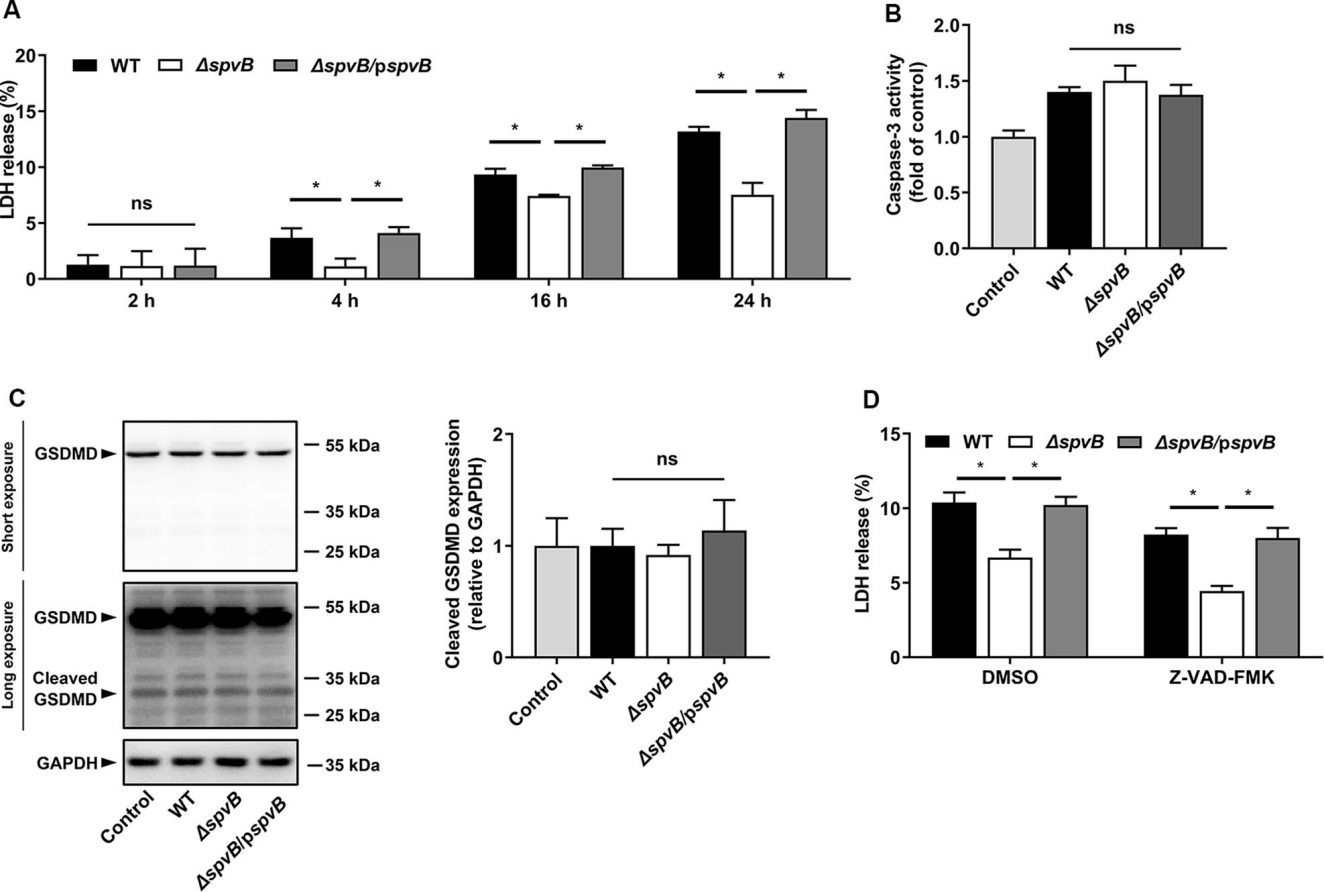
*Salmonella* pSLT-encoded effector SpvB induces caspase‐independent cell death. **A** Caco-2 cells were infected with the WT, *ΔspvB*, or *ΔspvB*/p*spvB S*. Typhimurium strain (MOI of 100) and incubated for 2, 4, 16, and 24 h. Aliquots of cellular supernatants were subjected to LDH release assay. **B**, **C** Caco-2 cells were infected with the WT, *ΔspvB*, or *ΔspvB*/p*spvB S*. Typhimurium strain (MOI of 100) and incubated for 4 h. **B** Cells were harvested and subjected to measurement of caspase-3 activity. **C** Western blot analysis of the expression of cleaved GSDMD. **D** Caco-2 cells were treated with either vehicle (DMSO) or 20 μM Z-VAD-FMK for 1 h. The cells were then infected with WT, *ΔspvB*, or *ΔspvB*/p*spvB S*. Typhimurium strain (MOI of 100) and incubated for 24 h. Aliquots of cellular supernatants were subjected to LDH release assay. Data were analyzed with IBM SPSS Statistics 19 and presented as the mean ± SEM using Student’s *t*-test and ANOVA with S-N-K correction. *P < 0.05; ns, not significant.

A number of genes that encode several important virulence effectors are located on the pSLT plasmid [4]. Among them, the *spv* is one of the most important virulence genes, and its encoded effector SpvB is crucial for the virulence phenotype of the *spv* locus [10]. To assess whether the *spvB* gene contributes to cell death, in the current study, human colon carcinoma cells (Caco-2) were infected with the wild-type (WT) *S*. Typhimurium or mutant strains lacking pSLT (*ΔpSLT*), *spv* (*Δspv*), and *spvB* (*ΔspvB*) respectively. By detecting the release of lactate dehydrogenase (LDH), an established indicator of cell death, we found that *ΔpSLT*-, *Δspv*-, and *ΔspvB*-infected cells showed a lower rate of cell death than the WT-infected cells (Supplemental Fig. 1A). Consistent with the observations from the LDH assays, we confirmed these findings using Ethidium Homodimer 1 (EthD-1), which is considered a DNA staining marker of dead cells (Supplemental Fig. 1B). These data suggested that the *Salmonella* pSLT-encoded effector SpvB has a crucial role in cell death. To confirm these observations and further investigate the dynamic effect of SpvB, we evaluated cell death at different time points after *Salmonella* infection. Although no significant difference in cell death was observed among Caco-2 cells infected with the WT, *ΔspvB*, or *spvB* complemented (*ΔspvB/*p*spvB*) strain at 2 h postinfection, a lower death rate was found in the *ΔspvB*-infected cells than in the WT- or the *ΔspvB/*p*spvB*-infected cells at 4, 16, and 24 h postinfection (Fig. 1A). Several types of RCD occurred during the stage of infection, including caspase-independent and caspase‐dependent RCD such as apoptosis and pyroptosis [11]. It has been reported that SpvB causes apoptotic cell death in eukaryotic cells [12]. However, in the *Salmonella*-infected Caco-2 cells, we found that SpvB deficiency had no effect on the activity of caspase-3, a key mediator of apoptosis (Fig. 1B). Recent studies have reported that *Salmonella* effectors could trigger pyroptosis [13]. To verify whether SpvB-induced cell death was associated with pyroptosis, we detected the cleavage of gasdermin D (GSDMD)—a crucial executor of pyroptosis. We found no significant difference among the WT-, *ΔspvB*-, or *ΔspvB/*p*spvB*-infected cells (Fig. 1C). To further determine whether cell death induced by SpvB is dependent on caspases, the pan‐caspase inhibitor Z-VAD-FMK was used; we found that downregulating caspase activity failed to block SpvB-induced cell death (Fig. 1D). Together, these data suggest that SpvB induces cell death in a caspase-independent manner.

### Result 2 SpvB promotes necroptosis of IECs

**Fig. 2:**
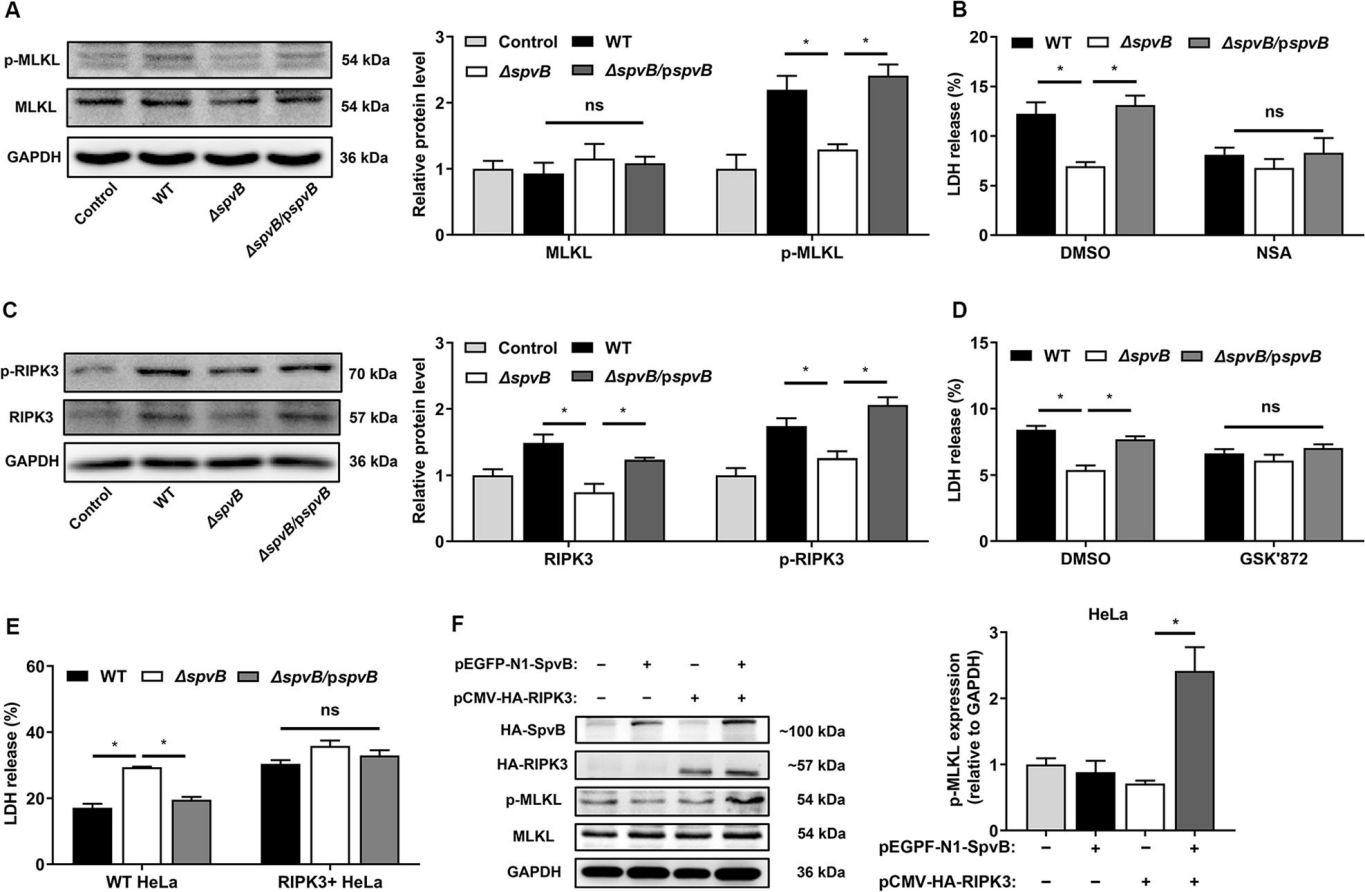
SpvB promotes necroptosis of IECs. **A**, **C** Caco-2 cells were infected with the WT, *ΔspvB*, or *ΔspvB*/p*spvB S*. Typhimurium strain (MOI of 100) and incubated for 4 h. Western blot analysis of the expression of **A** MLKL and p-MLKL (S358), **C** RIPK3 and p-RIPK3 (S227). **B**, **D** Caco-2 cells were treated with either vehicle (DMSO), **B** 1 μM necrosulfonamide (NSA) or **D** 1 µM GSK’872 for 1 h. The cells were then infected with the WT, *ΔspvB*, or *ΔspvB*/p*spvB S*. Typhimurium strain (MOI of 100) and incubated for 24 h. Aliquots of cellular supernatants were subjected to LDH release assay. **E** HeLa cells were transiently transfected with pCMV-HA-RIPK3 for 24 h. Cells were then infected with WT, *ΔspvB*, or *ΔspvB*/p*spvB S*. Typhimurium strain (MOI of 100) and cultured for 24 h. Aliquots of cellular supernatants were subjected to LDH release assay. **F** HeLa cells were transiently transfected with pCMV-HA-RIPK3 and pEGFP-N1-SpvB for 24 h. Western blot analysis of the expression of p-MLKL. Data were analyzed with IBM SPSS Statistics 19 and presented as the mean ± SEM using Student’s *t*-test and ANOVA with S-N-K correction. *P < 0.05; ns not significant.

Necroptosis is a form of caspase-independent RCD, which is associated with intestinal homeostasis and antibacterial defense [14]. Next, we focused on detecting the link between SpvB and necroptosis in IECs. Necroptosis occurs when p-MLKL oligomerizes, translocates to the plasma membrane and subsequently forms a lytic pore. In the *Salmonella* infected Caco-2 cells, we found that the phosphorylation of MLKL dramatically increased in the WT- and *ΔspvB*/p*spvB*-infected cells instead of *ΔspvB*-infected cells (Fig. 2A). As a complementary approach to confirm these findings, Caco-2 cells were pretreated with the MLKL inhibitor necrosulfonamide (NSA). We found that the treatment with NSA resulted in a decrease in SpvB-mediated cell death, and no significant difference was observed among cells infected with WT, *ΔspvB*, or *ΔspvB/*p*spvB* (Fig. 2B). The phosphorylation of RIPK3, an upstream molecule of MLKL, could recruit the MLKL and subsequently mediate its phosphorylation. As shown in Fig. 2C, the level of phospho-RIPK3 (p-RIPK3) was significantly higher in the WT- and the *ΔspvB/*p*spvB*-infected cells than in the *ΔspvB*-infected cells. GSK'872, an inhibitor targeting RIPK3, was used to confirm these findings; the results showed that GSK’872 eliminated the significant difference in cell death among the three infected groups (Fig. 2D). Human cervical carcinoma cells (HeLa), deficient in RIPK3 expression [15], were used to investigate the role of RIPK3 in SpvB-mediated cell death. Interestingly, the *ΔspvB*-infected cells showed a higher rate of cell death than the WT- and the *ΔspvB/*p*spvB*-infected HeLa cells, whereas the significant difference in cell death was eliminated in the case of RIPK3 overexpression (Fig. 2E). Moreover, western blot analysis showed that the level of MLKL phosphorylation was higher in cells co-transfected with pEGFP-N1-SpvB and pCMV-HA-RIPK3 plasmid, relative to that of the control (Fig. 2F). Taken together, we can conclude that SpvB promotes necroptosis during *Salmonella* infection.

### Result 3 SpvB promotes IECs necroptosis in a manner independent of RIPK1

**Fig. 3:**
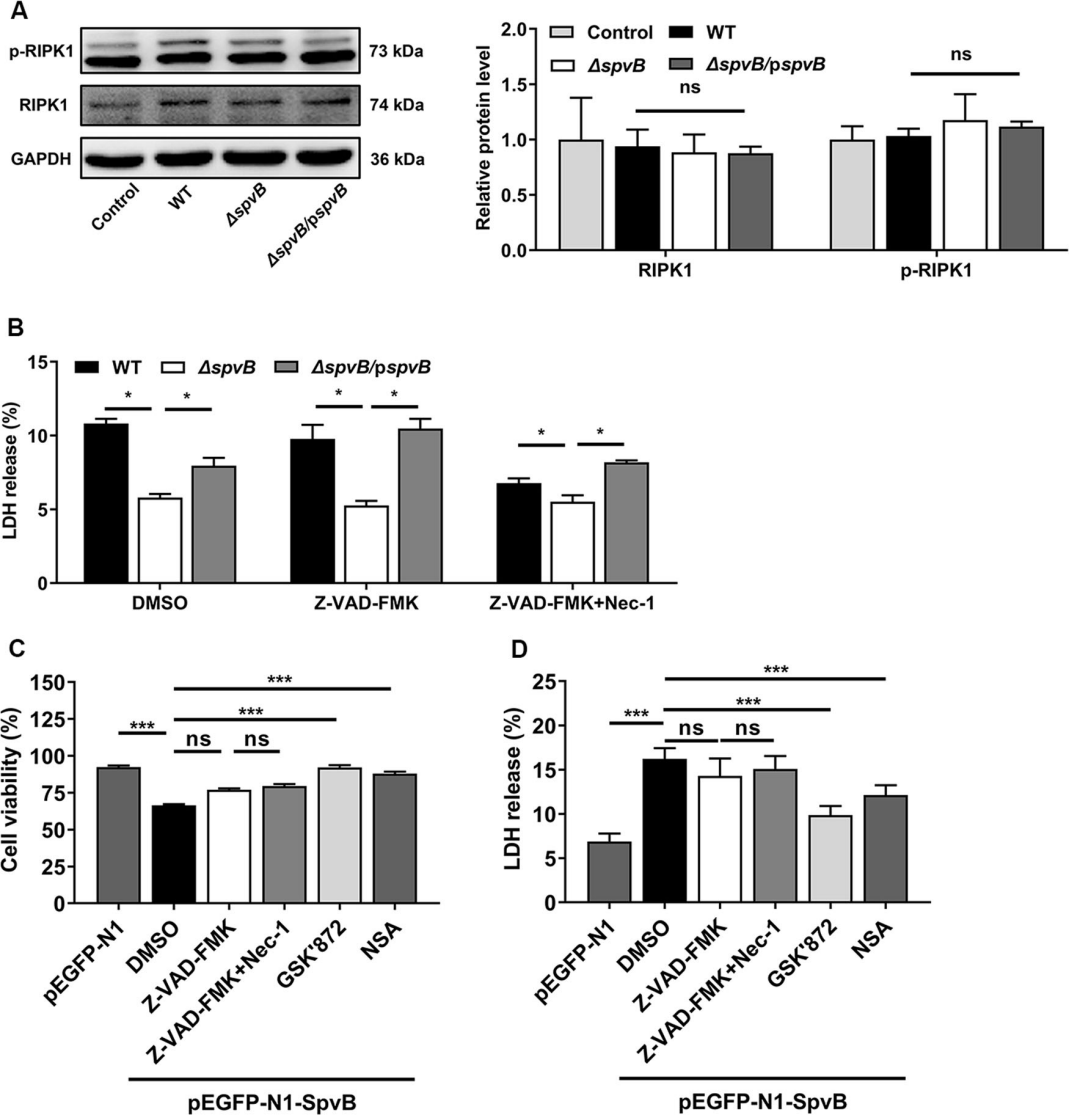
SpvB promotes IECs necroptosis in a manner independent of RIPK1. **A** Caco-2 cells were infected with WT, *ΔspvB*, or *ΔspvB*/p*spvB S*. Typhimurium strain (MOI of 100) and incubated for 4 h. Western blot analysis of the expression of RIPK1 and p-RIPK1. **B** Caco-2 cells were treated with either vehicle (DMSO), 20 µM Z-VAD-FMK with or without 10 µM necrostatin-1 (Nec-1) for 1 h. The cells were then infected with the WT, *ΔspvB*, or *ΔspvB*/p*spvB S*. Typhimurium strain (MOI of 100) and incubated for 24 h. Aliquots of cellular supernatants were subjected to LDH release assay. **C**, **D** Caco-2 cells were transiently transfected with pEGFP-N1 or pEGFP-N1-SpvB for 24 h. Cells transfected with pEGFP-N1-SpvB were pretreated with either vehicle (DMSO), 20 µM Z-VAD-FMK with or without 10 µM Nec-1, 1 µM GSK’872, or 1 µM NSA for 1 h. **C** Cells were subjected to cell viability assay. **D** Aliquots of cellular supernatants were subjected to LDH release assay. Data were analyzed with IBM SPSS Statistics 19 and presented as the mean ± SEM using Student’s *t*-test and ANOVA with S-N-K correction. *P < 0.05, ***P < 0.001; ns not significant.

Receptor-interacting protein kinase 1 (RIPK1) is a key molecule in mediating necroptosis, which recruits RIPK3 and leads to its phosphorylation. However, the activation of RIPK3 could also occur in a manner independent of RIPK1 [9]. To determine whether RIPK1 is involved in SpvB-mediated necroptosis, we measured phospho-RIPK1 (p-RIPK1) level in Caco-2 cells after *Salmonella* infection. Western blot analysis showed no significant differences in the expression levels of RIPK1 and p-RIPK1 among cells infected with WT, *ΔspvB*, or *ΔspvB/*p*spvB* (Fig. 3A). To further confirm these observations, cells were treated with the RIPK1 inhibitor necrostatin-1 (Nec-1) with or without the Z-VAD-FMK. In line with the data presented in Fig. 1D, the death rate was still lower in cells infected with *ΔspvB* than infected with WT and *ΔspvB/*p*spvB* following solely employing Z-VAD-FMK. Importantly, treatment with Nec-1 and Z-VAD-FMK reduced *Salmonella*-induced cell death but failed to reverse the effect of SpvB in this biologic process (Fig. 3B). These data imply that SpvB induces necroptosis in a RIPK1-independent manner. To further prove these observations, we performed transfection experiments using pEGFP-N1-SpvB overexpression plasmid [12]. In line with the findings obtained in *Salmonella*-infected Caco-2 cells, the overexpression of SpvB resulted in a significant increase in cell death which could not be inhibited by Z-VAD-FMK. Treatment with either NSA or GSK’872 significantly reduced SpvB-mediated cell death, whereas no significant difference was observed between Nec-1-treated cells and corresponding control cells (Fig. 3C, D). These findings suggest that SpvB-mediated necroptosis is independent of RIPK1.

### Result 4 SpvB leads to epithelium necroptosis through inhibiting RIPK3 ubiquitination and autophagy-dependent degradation

**Fig. 4:**
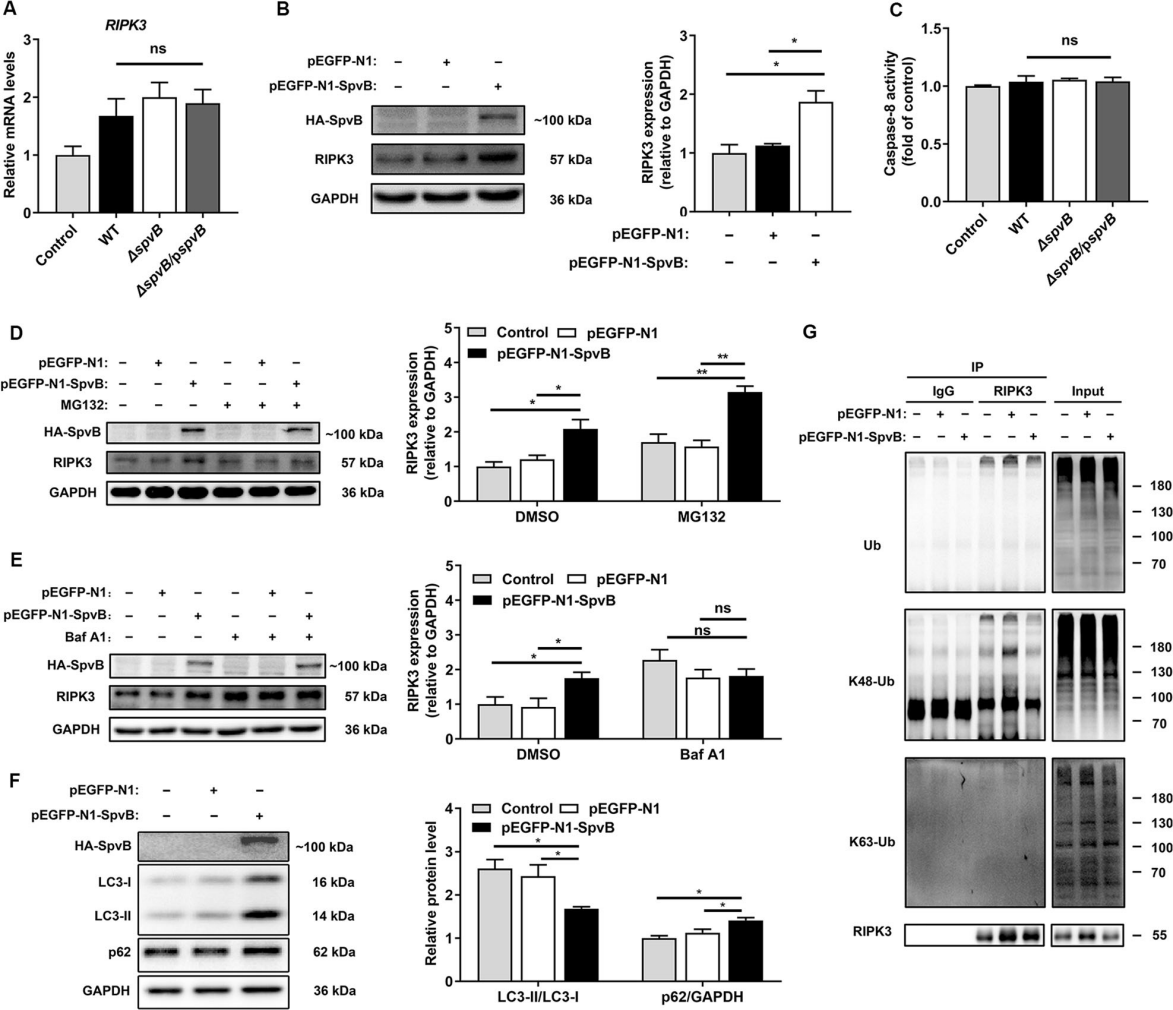
SpvB leads to epithelium necroptosis through inhibiting RIPK3 ubiquitination and autophagy-dependent degradation. **A**, **C** Caco-2 cells were then infected with WT, *ΔspvB*, or *ΔspvB*/p*spvB S*. Typhimurium strain (MOI of 100) and incubated for 4 h. **A** *RIPK3* levels were determined by RT-qPCR. Values were normalized to those of the housekeeping gene *β‐ACTIN* and fold‐changes relative to the untreated control were shown. **C** Cells were harvested and subjected to measurement of caspase-8 activity. **B**, **D**–**G** Caco-2 cells were transiently transfected with pEGFP-N1-SpvB or pEGFP-N1 for 24 h. **B** Western blot analysis of the expression of RIPK3. **D** Cells were treated with 5 μM MG132 for 24 h and western blot analysis of the expression of RIPK3. **E** Cells were treated with 100 nM bafilomycin A1 (Baf A1) and western blot analysis of the expression of RIPK3. **F** Western blot analysis of the expression of LC3-I, LC3-II, and p62. **G** Cell lysates were immunoprecipitated with anti-RIPK3 antibody or IgG, then immunoblotted with respective antibodies. Data were analyzed with IBM SPSS Statistics 19 and presented as the mean ± SEM using Student’s *t*-test and ANOVA with S-N-K correction. *P < 0.05, **P < 0.005; ns not significant.

Given that RIPK3 is reserved predominantly for necroptosis in the IECs [16–18], we further investigated the molecular mechanism underlying SpvB-associated RIPK3 abundance observed in Fig. 2C. We first explored whether SpvB regulates the RIPK3 transcription and found no significant difference in the mRNA levels of *RIPK3* among the WT-, *ΔspvB-*, and *ΔspvB*p*spvB*-infected groups (Fig. 4A). Western blot analysis showed that the cells transfected with SpvB displayed an increased expression level of RIPK3 (Fig. 4B). Accordingly, we focused on elucidating the post-translational modification of RIPK3 by SpvB. Caspase-8-mediated RIPK3 cleavage is a physiological approach to downregulating the level of necroptosis [19, 20]. However, we did not detect a significant difference in caspase-8 activity among the three strains of *Salmonella* infection (Fig. 4C). It has been reported that RIPK3 protein can undergo rapid degradation by proteasome and autophagolysosome [17, 21, 22]. MG132, an inhibitor of proteasome, failed to reverse SpvB-mediated RIPK3 accumulation (Fig. 4D). Interestingly, treatment with bafilomycin A1 (Baf A1)—an inhibitor of autophagy flux—resulted in no significant difference between cells transfected with pEGFP-N1 and pEGFP-N1-SpvB, suggesting a crucial role for autophagy in the regulation of RIPK3 degradation (Fig. 4E). To further confirm these findings, we detected the ratio of LC3-II/LC3-I and the expression level of p62, two well-known autophagy biomarkers [23]. Western blot analysis showed that the cells ectopically expressing SpvB displayed an increased expression level of p62 and a decreased ratio of LC3-II/LC3-I (Fig. 4F). Ubiquitination is a versatile post-translational modification that determines selectivity in autophagy [24]. As shown in Fig. 4G, the ubiquitination of RIPK3 was decreased in the SpvB-transfected cells. We further detected the levels of K48- and K63-type polyubiquitination of RIPK3 in these transfected cells. Importantly, we found that K48-linked polyubiquitination, but not K63-linked polyubiquitination, was considerably lower in the presence of SpvB (Fig. 4G). Collectively, these data suggest that SpvB restrains RIPK3 K48-linked polyubiquitination, thus downregulating RIPK3 degradation in an autophagy-dependent manner.

### Result 5 SpvB promotes the pathogenicity of *Salmonella* and induces colon cell death in vivo

**Fig. 5:**
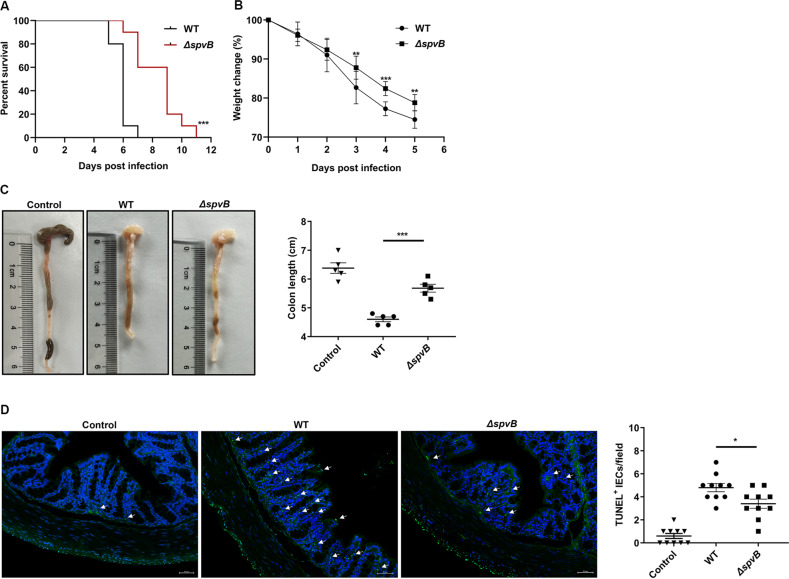
SpvB promotes the pathogenicity of *Salmonella* and induces colon cell death in vivo. **A**, **B** C57BL/6 mice were treated with streptomycin 24 h prior to oral infection with the WT or *ΔspvB S*. Typhimurium strain (1 × 10^7^ CFUs). **A** Survival curves, *n* = 10 mice per group. **B** Body weight changes, *n* = 10 mice per group. **C**, **D** C57BL/6 mice were treated with streptomycin 24 h prior to oral infection with the WT or *ΔspvB S*. Typhimurium strain (1 × 10^8^ CFUs) and analyzed at 48 h. **C** Colon length and representative photographs of colons, *n* = 5 mice per group. **D** Immunofluorescence staining of colon sections from mice and representative images of TUNEL-positive cells were denoted by arrows, counted ten high-power fields (TUNEL, green; DAPI, blue). Scale bars: 50 μm. Survival curves were analyzed with the log-rank test. Data were analyzed with IBM SPSS Statistics 19 and presented as the mean ± SEM using Student’s *t*-test. *P < 0.05, **P < 0.005, ***P < 0.001; ns not significant.

Our in vitro studies suggested that SpvB promoted IECs necroptosis by regulating the degradation of RIPK3, so we next sought to investigate whether SpvB contributes to promoting the pathogenicity of *Salmonella* and inducing cell death in vivo. Mice were infected with the WT or *ΔspvB S*. Typhimurium strains, and we found the WT-infected mice displayed a higher mortality and lower body weight relative to the *ΔspvB*-infected mice (Fig. 5A, B). These observations suggested that SpvB aggravated the severity of salmonellosis. Compared with *ΔspvB*-infected mice, WT-infected mice had a decreased colon length, which is indicative of severe colitis (Fig. 5C). TUNEL staining is a nonspecific method for evaluating cell death that detects DNA fragments [25]. As shown in Fig. 5D, the number of TUNEL-positive epithelial cells in *the ΔspvB*-infected colon was lower than that in the WT-infected colon. These observations demonstrate that SpvB promotes intestinal injury and cell death in colonic IECs of mice.

### Result 6 SpvB promotes necroptosis of IECs in vivo

**Fig. 6:**
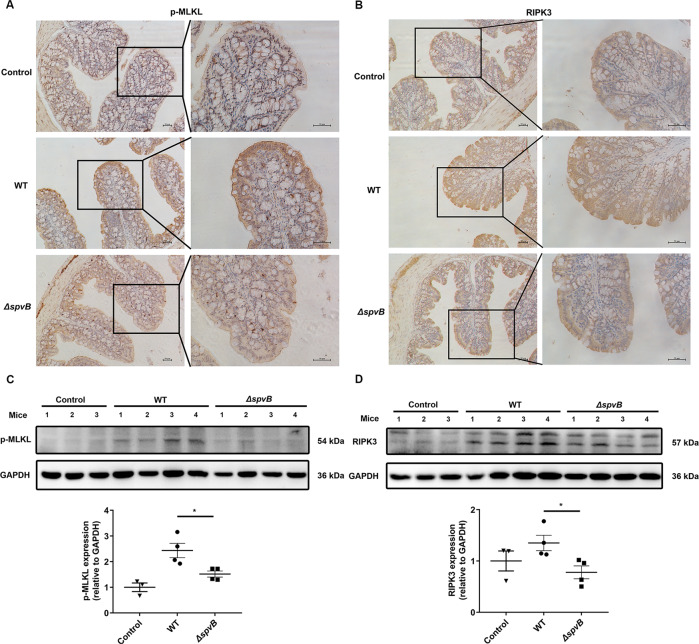
SpvB promotes necroptosis of IECs in vivo. **A**, **B** C57BL/6 mice were treated with streptomycin 24 h prior to oral infection with the WT or *ΔspvB S*. Typhimurium strain (1 × 10^8^ CFUs) and analyzed at 48 h. IHC staining evaluated the level of **A** p-MLKL and **B** RIPK3 in colon sections. Scale bars: 50 μm. **C**, **D** Western blot analysis of purified colonic epithelial lysates with specific antibodies to **C** p-MLKL and **D** RIPK3, *n* = 3 mice for control group, *n* = 4 mice for WT- and *ΔspvB-*infected group respectively. Data were analyzed with IBM SPSS Statistics 19 and presented as the mean ± SEM using Student’s *t*-test. *P < 0.05.

To investigate the relationship between SpvB and colonic necroptosis, the levels of p-MLKL and RIPK3 were examined by immunohistochemistry. We observed that the expression of p-MLKL was much higher in the colon of the WT-infected mice than that in the *ΔspvB*-infected mice (Fig. 6A). We also found an abundant accumulation of RIPK3 in the colon of WT-infected mice (Fig. 6B). We further isolated IECs from the colon of WT- and *ΔspvB*-infected mice. Western blot analysis showed that WT-infected IECs displayed a higher phosphorylated level of MLKL than *ΔspvB*-infected mice. Consistently, we found a significant upregulation of RIPK3 in the WT-infected IECs (Fig. 6C, D). Taken together, these findings demonstrate that SpvB promotes necroptosis of IECs in vivo.

## Discussion

**Fig. 7:**
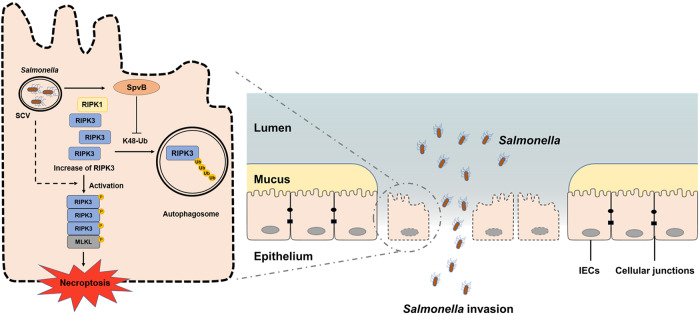
General summary of SpvB-mediated IECs necroptosis during *Salmonella* infection. SpvB, an effector encoded by pSLT, downregulates the K48-Ub-degradation of RIPK3 in an autophagy-dependent manner. The abundant accumulation of RIPK3 upregulates the phosphorylation level of MLKL and leads to necroptosis in IECs. The SpvB-mediated IECs necroptosis aggravates the pathogenesis of *Salmonella* through promoting the bacteria to disrupt intestinal epithelial barrier integrity. IECs, intestinal epithelial cells; SCV, *Salmonella*-containing vacuole; MLKL, mixed-lineage kinase domain-like protein; RIPK3, receptor-interacting protein kinase 3; RIPK1, receptor-interacting protein kinase 1.

*Salmonella* spreads to both humans and animals through the fecal–oral route. The gastrointestinal tract is the first site of host–pathogen interaction after ingestion of *Salmonella*. IECs exert an influence on maintaining intestinal mucosal barrier function to resist the invasion by *Salmonella*. Our previous studies have reported that SpvB promotes *S*. Typhimurium intracellular replication by interfering with the host’s iron homeostasis [26, 27]. In the current study, we further showed that SpvB contributes to the induction of necroptosis through downregulating K48-Ub-mediated degradation of RIPK3, which is ultimately advantageous for *Salmonella* across the intestinal epithelial barrier (Fig. 7).

The intestinal mucosal barrier has functions to separate intestinal lumen material, resist pathogen invasion, and maintain the homeostasis of the organism [28, 29]. As the structural basis of the intestinal mucosal barrier, the epithelial barrier including IECs and intercellular junctions is of great significance for the host to resist the invasion by *Salmonella*. Our findings demonstrated that SpvB promoted the pathogenicity of *Salmonella*, including an increase in mortality and a decrease in body weight. We also found that SpvB resulted in a decrease in colon length and induced cell death in the colon. We further constructed the Caco-2 cells infection model to confirm these observations in vitro. Hanna et al. suggested that SpvB-delayed cell death may occur at a later stage and cannot be captured [30, 31]. Interestingly, in this study, we found that SpvB induced IECs cell death, and the existence of SpvB-associated cell death was captured at an early phase during *Salmonella* infection.

The pSLT-encoded SpvB has been shown to contain an ADP-ribosyltransferase domain in its C-terminus. Some researchers believe that the type of cell death induced by SpvB is apoptosis [30, 31], which is a complex multistep process driven by caspase-dependent proteolytic cleavage cascades [32]. However, the SpvB deficiency had no effect on the apoptosis mediator caspase-3 in our study. Moreover, the treatment with the pan-caspase inhibitor Z-VAD-FMK had no significant influence on SpvB-mediated cell death. These observations consistent with early research suggested that SpvB-associated cell death is an unknown type of RCD [12]. Necroptosis, which has morphological characteristics similar to necrosis, is typically considered a highly pro-inflammatory mode of cell death due to the release of damage-associated molecular patterns [33]. Therefore, this type of RCD plays a role in many intestinal diseases associated with inflammation, such as inflammatory bowel disease and necrotizing enterocolitis [34–36]. Necroptosis of the IECs often leads to uncontrolled translocation of the bacteria and excessive inflammation [37]. Interestingly, we found that SpvB induced the phosphorylation of MLKL and RIPK3, suggesting an increase in necroptosis. By employing various inhibitors, we further confirmed these observations. RIPK1, a key molecule, can phosphorylate RIPK3 and then induce necroptosis [9]. However, the inhibitor of RIPK1 failed to eliminate the significant effect of the SpvB on cell death. These results demonstrate that SpvB mediates the increased necroptosis in IECs in a RIPK3-dependent manner.

Previous investigations have demonstrated that RIPK3 accumulation correlates with severe IECs necroptosis and colitis [16, 17, 38]. Interestingly, we found that the protein level of RIPK3 was substantially increased in the presence of SpvB both in vivo and in vitro. These observations suggested that the RIPK3 overexpression is the basis for SpvB to promote necroptosis. RIPK3 is a critical mediator of necroptosis and could be regulated at several levels. Previous studies have shown that caspase-8 could cleave RIPK3 to inhibit necroptosis of IECs during the process of enteritis caused by *Salmonella*, thereby playing a role in maintaining the intestinal barrier function and limiting the colonization of pathogens [19, 20]. In some diseases, such as colon cancer and Cowpox virus infection, the expression of RIPK3 could be regulated through transcription mechanisms [39, 40]. At the protein level, RIPK3 could also be regulated through post-transcriptional modification, such as autophagy and proteasome [17, 21, 22]. In the current study, SpvB had no obvious effect on the activity of caspase-8. We also detected no significant effect of SpvB on the RIPK3 transcription level in this infection model. Thus, we focused on the hypothesis that SpvB-mediated increase in RIPK3 protein might indicate an inactive degradation mechanism. In line with our hypothesis, treatment with the autophagy inhibitor Baf A1 eliminated the effects of SpvB on RIPK3 and the K48-ubiquitination of RIPK3 was increased in the presence of SpvB. Taking comprehensive consideration of Fig. 4, these results are consistent with our assumption that SpvB upregulates necroptosis through inhibiting the K48-Ub-dependent autophagy degradation of RIPK3.

In summary, we found a novel function of the effector SpvB to aggravate the pathogenesis of *Salmonella* through inducing IECs necroptosis, thereby promoting the bacteria to disrupt the integrity of the intestinal epithelial barrier. By downregulating the K48-Ub-degradation of RIPK3 in an autophagy-dependent manner, SpvB mediates the abundant accumulation of RIPK3. The accumulation of RIPK3 increases the phosphorylation level of MLKL and upregulates necroptosis in IECs. The disturbed integrity of IECs ultimately leads to the disruption of the intestinal epithelial barrier and aggravated *Salmonella* infection. In vivo, SpvB promotes salmonellosis, manifested as increased mortality, decreased body weight, and severe colon injury. Taken together, *Salmonella* effector SpvB induces IECs necroptosis in the early stage of the infection, which may be considered the strategy of bacterial invasion.

## Materials and methods

### Cell culture

Caco-2 cells were kindly provided by Professor Weiqi He (Soochow University, Suzhou, China). HeLa cells were purchased from the American Type Culture Collection (Manassas, VA, USA). The obtained cells were cultured in Dulbecco’s Modified Eagle’s medium (DMEM; HyClone Laboratories, Logan, UT, USA) with 10% fetal bovine serum (FBS; Biological Industries, Kibbutz Beit‐Haemek, Israel) and 1% penicillin–streptomycin (Beyotime Biotechnology, Shanghai, China) solution at a temperature of 37 °C and 5% CO_2_.

### Bacterial strains and growth conditions

The wild-type (WT) *S*. Typhimurium strain SL1344, mutants lacking *spv* (*Δspv*) or *spvB* (*ΔspvB*) and *spvB* complemented (*ΔspvB*/p*spvB*) in plasmid pBAD/gIIIA were used in the study as previously described [26]. The mutant lacking *pSLT* was constructed by plasmid elimination assay with sodium dodecyl sulfate (SDS) treatment and checked by means of PCR. For SDS treatment, 30 μL of a bacterial suspension was added to 3 mL of 10% SDS Luria‐Bertani (LB) broth (Hangwei, Hangzhou, China) and incubated at 37 °C with shaking overnight. A total of 30 μL of the bacterial suspension treated with SDS was added to 3 mL LB broth and incubated at 37 °C with shaking for 14–16 h. After repeating the above two steps several times, 30 μL of the bacterial suspension was grown on LB agar (Hangwei), and the elimination of the plasmid was identified by PCR with specific primers. Bacteria were grown on LB agar plates and then in LB broth at 37 °C with shaking for 14–16 h. Bacteria were diluted 1:100 with fresh LB medium supplemented with 100 µg/mL ampicillin when appropriate and cultured until the logarithmic phase was reached.

### *Salmonella* infection in vitro

The bacteria reaching the late‐logarithmic phase were washed three times with phosphate buffer saline (PBS) and then quantified using a spectrophotometer to determine optical density at 600 nm and calculate the multiplicity of infection (MOI). Caco-2 and HeLa cells were infected with bacteria at MOI of 100 in DMEM-10% FBS (v/v). One hour later, the cells were washed twice with warm PBS, and the medium was replaced with DMEM-10% FBS (v/v) containing 100 µg/mL amikacin (MilliporeSigma, Burlington, MA, USA) to prevent the growth of extracellular bacteria for 2 h. Afterward, the infected cells were washed and subsequently incubated with DMEM-FBS (10%) and 10 µg/mL amikacin. Cells were subsequently treated with DMSO (MilliporeSigma) vehicle, 5 µM MG123 (Selleck Chemicals, Houston, TX, USA) or 100 nM bafilomycin A1 (Baf A1; MCE) when appropriate.

### *Salmonella* infection in vivo

Female C57BL/6 mice (6–8 weeks) were bred and maintained at the experimental animal center of Soochow University. All animal experiments were approved by the Ethics Committee of Soochow University, Suzhou, China, and were conducted in accordance with the Guidelines for the Care and Use of Research Animals established by Soochow University. For experiments of mouse survival and body weight, the mice were treated with streptomycin (0.1 mL of a 200 mg/mL solution in sterile water) 24 h prior to oral infection with 1 × 10^7^ colony forming units (CFUs) of different *S*. Typhimurium strains. For experiments that require the organs of mice, the mice were treated with streptomycin (0.1 mL of a 200 mg/mL solution in sterile water) 24 h prior to oral infection with 1 × 10^8^ CFUs of different *S*. Typhimurium strains and euthanized 48 h postinfection using CO_2_ asphyxiation. The control mice received only PBS.

### Immunoblot analysis

Cells were washed once with PBS, followed by lysis in RIPA buffer (Beyotime Biotechnology) containing protease and phosphatase inhibitors (Beyotime Biotechnology) and sample loading buffer. Proteins were separated by electrophoresis on 8–12% polyacrylamide SDS-PAGE gels and transferred onto polyvinylidene difluoride membranes (MilliporeSigma). Nonspecific binding was blocked with either 5% nonfat dry milk powder or 5% bovine serum albumin in Tris-buffered saline containing 0.1% Tween 20. Membranes were probed with primary antibodies at 4 °C overnight, then washed and incubated with the appropriate horseradish peroxidase-conjugated secondary antibodies including anti-rabbit IgG (ab97051, Abcam, Cambridge, MA, USA) and anti-mouse IgG (Beyotime Biotechnology) at room temperature for 1 h. Proteins were visualized using an enhanced chemiluminescence luminescence reagent (Meilunbio, Dalian, China). The intensities of the target bands were quantified using ImageJ Launcher broken symmetry software program (National Institutes of Health, Bethesda, MD, USA). The antibodies used were as following: anti-GSDMD (ab210070, Abcam); anti-MLKL (YT2788, Immunoway, Plano, TX, USA); anti-p-MLKL (phospho S358) (ab187091, Abcam); anti-p-MLKL (phospho S345) (ab196436, Abcam); anti-RIPK3 (17563-1-AP, Proteintech, Rosemont, IL, USA); anti-p-RIPK3 (phospho S227) (ab209384, Abcam); anti-p-RIPK1 (phospho S166) (YP1467, Immunoway); anti-RIPK1 (17519-1-AP, Proteintech); anti-K63-Ubiquitin (ab179434, Abcam); anti-K48-Ubiquitin (ab140601, Abcam); anti-Ubiquitin (#3936, Cell Signaling Technology, Danvers, Massachusetts, USA); anti-HA (#3724, Cell Signaling Technology); anti-GAPDH (BA2913, Boster, Wuhan, China); anti-LC3 (NBP100-2220, Novus Biologicals, Littleton, Colorado, USA); anti-p62 (#5114, Cell Signaling Technology).

### Cell transfection

HeLa cells were transiently transfected with pEGFP-N1-SpvB (fusion protein with HA tag) or pCMV-HA-RIPK3 using ExFect2000 Transfection Reagent (Vazyme, Nanjing, China) for 24 h in accordance with the manufacturer’s instructions. Caco-2 cells were transiently transfected with pEGFP-N1-SpvB or pEGFP-N1.

### Immunohistochemistry (IHC) and TUNEL assays

After the mice were euthanized, detached colons were fixed in 10% formalin, then processed and embedded in paraffin in line with the standard procedures. Formalin-fixed paraffin-embedded colons were cut into 5-μm-thick sections. IHC staining was performed using antibody directed against p-MLKL (phospho S345) and RIPK3. TUNEL staining was performed using the Dead-End kit (Promega, Madison, WI, USA) in accordance with the manufacturer’s instructions.

### IECs isolation

IECs were isolated as described previously [41]. In brief, the colons from the mice were opened longitudinally and washed in a solution containing 0.154 M NaCl and 1 mM DTT to remove intestinal contents, and then incubated in a solution containing 1.5 mM EDTA and 1 mM DTT at 37 °C for 30 min. IECs were obtained by centrifugation for subsequent protein extraction.

### Lactate dehydrogenase (LDH) and cell viability assays

Cells were seeded onto 96-well plates at a density of 30,000 cells per well. The second day after seeding, the cells were counted and pretreated with 20 μM Z-VAD-FMK (Beyotime Biotechnology), 1 μM necrosulfonamide (MCE, Monmouth Junction, NJ, USA), 1 μM GSK’872 (MCE), 10 μM necrostatin-1 (MCE), or DMSO vehicle control for infection or transfection. LDH released was determined by the LDH cytotoxicity assay detection kit (Beyotime Biotechnology). The cell culture supernatant was collected for detection. The absorbance was read at 490 nm with Infinite^®^ F50 Absorbance Microplate Reader (Tecan, Switzerland). Cell viability was measured based on the intracellular ATP levels using CellTiter-Lumi™ Plus Luminescent Cell Viability Assay Kit (Beyotime Biotechnology). An equal volume of Celltiter-LUMI™ Plus reagent was added to the cell culture medium to induce cell lysis by oscillation. After incubation at room temperature, the luminescence signal was measured by Synergy^TM^ 2 Multi-Mode Microplate Reader (BioTek, Winooski, VT, USA).

### Quantitative real-time PCR (qRT-PCR)

Total RNA was isolated from Caco-2 cells with trizol (Thermo Fisher Scientific, Waltham, MA, USA) reagent and subjected to reverse transcription using the All-in-one RT MasterMix kit (Applied Biological Materials, Richmond, BC, Canada). qRT-PCR was conducted using the ViiA7 real-time PCR instrument (Applied Biosystems, Carlsbad, CA, USA) with EvaGreen MasterMix-Low ROX (Applied Biological Materials) to analyze transcript levels of target genes. The expression level of *RIPK3* was normalized to *β-ACTIN* expression with the 2^−ΔΔCT^ method. Each sample was detected in triplicate. The primer sequences for *RIPK3* and *β-ACTIN* were as follows: *hRIPK3*, 5'-GCTACGATGTGGCGGTCAAGAT-3' and 5'-TTGGTCCCAGTTCACCTTCTCG-3' [42]; *hβ-ACTIN*, 5'-CACCATTGGCAATGAGCGGTTC-3' and 5'-AGGTCTTTGCGGATGTCCACGT-3'.

### Caspase activity assay

Caco-2 cells were plated in each well of a 12‐well plate and stimulated for 4 h with the indicated *S*. Typhimurium strains at MOI of 100. Caspase-3 and -8 activities in cell lysates were analyzed using Caspase-3 and Caspase-8 Activity Assay Kit (Beyotime Biotechnology) respectively in accordance with the manufacturer’s protocol.

### Immunoprecipitation

Cells were washed once with PBS, followed by lysis in RIPA buffer (Beyotime Biotechnology) containing protease and phosphatase inhibitors. The whole-cell lysates were cleared for 30 min with 1 µg of rabbit IgG and 20 µL of Protein A/G PLUS-Agarose (Santa Cruz Biotechnology, Dallas, TX, USA). After pellet beads were removed by centrifugation, the cell lysates were treated with 1 µg anti-RIPK3 antibody (17563-1-AP, Proteintech) for 1 h, followed by incubation with pellet beads on a shaker overnight. Immunoprecipitated proteins and pellet beads were collected by centrifugation for subsequent experiments.

### Ethidium homodimer 1 (EthD-1) assay

Caco-2 cells were plated onto glass coverslips and then infected with respective bacteria at MOI of 100 for 24 h. In accordance with the manufacturer’s instructions, EthD-1 (US EVERBRIGHT INC., Suzhou, China) was diluted to 4 μΜ in PBS and incubated with the cells for 40 min at room temperature. Then, the cells were incubated with DAPI (MilliporeSigma) for 15 min at room temperature and observed under a Nikon Eclipse Ni-U fluorescence microscope (Nikon, Tokyo, Japan). The percentage of the EthD-1-positive cells was then determined.

### Statistical analysis

Statistical analysis was performed using SPSS Statistics 19 software (IBM, Armonk, NY, USA). Survival curves were analyzed with log-rank (Mantel–Cox) test. The Student’s *t*-test was used for data comparison between two groups and one-way analysis of variance (ANOVA) with S-N-K correction was used for comparison between multiple groups after normality tests. These data were expressed as mean ± SEM, and *P* < 0.05 was considered to be statistically significant. Values indicate the number of animals used for the experiments. No statistical methods were used to predetermine sample sizes, but our sample sizes are similar to those reported in previous publications [7]. No randomization was used for the data collection.

## Supplementary information

Supplement Figure legend

Supplemental Figure1

## Data Availability

The datasets used and/or analyzed during the current study are available from the corresponding author on reasonable request.
