# Supplementary material for: Salmonella pSLT-encoded effector SpvB promotes RIPK3-dependent necroptosis in intestinal epithelial cells
Source: Cell Death Discov. 2022 Feb 2;8:44. doi: 10.1038/s41420-022-00841-9 (PMC8810775; doi:10.1038/s41420-022-00841-9)
Supplement: Supplementary file 1 — Supplement Figure legend [file 41420_2022_841_MOESM1_ESM.docx]

**Supplement figure 1 *Salmonella* pSLT-encoded effector SpvB induces cell death**

Caco-2 cells were infected with WT, *ΔpSLT*, *Δspv* or *ΔspvB* *S*. Typhimurium strain (MOI of 100) and incubated for 24 h. (A) Aliquots of cellular supernatants were subjected to LDH release assay. (B) Ethidium Homodimer 1 (EthD-1) staining of Caco-2 cells and representative images of positive cells were shown (EthD-1, red; DAPI, blue). Scale bars: 50 μm. Data were analyzed with IBM SPSS Statistics 19 and presented as the mean ± SEM using Student’s *t* test. ***P*＜0.01, ****P*＜0.001.
