# Supplementary figures and images for: Salmonella pSLT-encoded effector SpvB promotes RIPK3-dependent necroptosis in intestinal epithelial cells
Source: Cell Death Discov. 2022 Feb 2;8:44. doi: 10.1038/s41420-022-00841-9 (PMC8810775; doi:10.1038/s41420-022-00841-9)

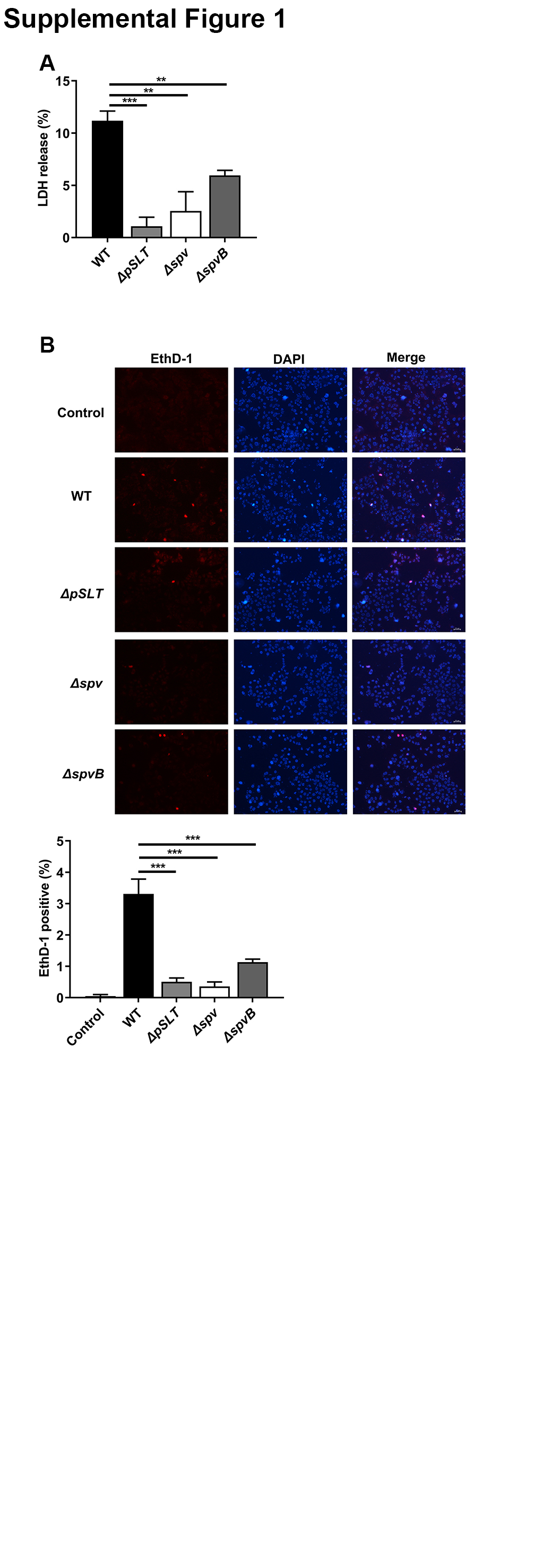

Supplement: Supplementary file 2 — Supplemental Figure1 [file 41420_2022_841_MOESM2_ESM.jpg]
